# Binding activity and specificity of tail fiber protein 35Q for *Salmonella* pullorum

**DOI:** 10.3389/fmicb.2024.1429504

**Published:** 2024-06-25

**Authors:** Hewen Deng, Linwan Feng, Kun Shi, Rui Du

**Affiliations:** ^1^College of Animal Science and Technology, Jilin Agricultural University, Changchun, China; ^2^College of Chinese Medicine Materials, Jilin Agricultural University, Changchun, China

**Keywords:** *Salmonella* pullorum, bacteriophage, tail fiber protein, bacterial detection, *Salmonella* bacteriophage

## Abstract

*Salmonella*, a prevalent pathogen with significant implications for the poultry industry and food safety, presents a global public health concern. The rise in antibiotic resistance has exacerbated the challenge of prevention. Accurate and sensitive detection methods are essential in combating *Salmonella* infections. Bacteriophages, viruses capable of targeting and destroying bacteria, leverage their host specificity for accurate microbial detection. Notably, the tail fiber protein of bacteriophages plays a crucial role in recognizing specific hosts, making it a valuable tool for targeted microbial detection. This study focused on the tail fiber protein 35Q of *Salmonella* pullorum (SP) bacteriophage YSP2, identified through protein sequencing and genome analysis. Bioinformatics analysis revealed similarities between 35Q and other *Salmonella* bacteriophage tail fiber proteins. The protein was successfully expressed and purified using an *Escherichia coli* expression system, and its binding activity and specificity were confirmed. ELISA assays and adsorption experiments demonstrated that 35Q interacts with the outer membrane protein (OMP) receptor on bacterial surfaces. This investigation provides valuable insights for targeted *Salmonella* detection, informs the development of specific therapeutics, and enhances our understanding of the interaction between *Salmonella* bacteriophages and their hosts.

## 1 Introduction

Pullorum is a bacterial infectious disease caused by *Salmonella* pullorum (SP) in chicks (Li et al., 2022). The disease can be transmitted vertically through eggs or horizontally among chicks (Barrow and Neto, 2011) through the respiratory and digestive tract (Lutful Kabir, 2010). It can cause systemic diseases in chicks and poses a serious threat to the poultry breeding industry. *Salmonella* is an animal disease pathogen under key surveillance and must be eliminated (Eng et al., 2015). In the early 20th century, *Salmonella* pullorum and Typhoid serotypes were significant pathogens causing epidemics in chicken flocks in Europe and the United States (Barrow and Neto, 2011; World Health Organization, 2016a; Marmion et al., 2021), especially in chicks under 4 weeks old (Barrow and Neto, 2011). The high infection and mortality rates seriously impact the hatchability and survival rates of breeder chickens, leading to substantial economic losses in the poultry breeding industry (Nair and Kollanoor Johny, 2019).

*Salmonella* encompasses over 2,500 different serotypes globally (Branchu et al., 2018; Ferrari et al., 2019), with more than 40 linked to poultry (El-Tawab et al., 2015) and the rest found in various animal species. These zoonotic pathogens can infect hosts across species (Stevens and Kingsley, 2021), causing symptoms and diseases. Within livestock and poultry populations, some animals act as latent carriers, intermittently transmitting the bacteria and serving as primary infection sources (Griffith et al., 2019). *Salmonella* spreads easily through direct or indirect contact between animals, humans (Shaji et al., 2023), and even via aerosols. The primary mode of infection is through the digestive tract, although aerosol transmission is also possible. Factors such as poor hygiene, stress, and concurrent viral or parasitic infections can trigger salmonellosis (Kemal, 2014). In humans, *Salmonella* can lead to typhoid fever, paratyphoid fever, acute gastroenteritis, and in severe cases, sepsis (Kemal, 2014). Young animals are particularly susceptible to *Salmonella*, experiencing sepsis, gastroenteritis, tissue inflammation, and potential miscarriages in pregnant individuals (Xie et al., 2021). Human ingestion of *Salmonella*-contaminated food can result in acute food poisoning (Sa and La, 2019), highlighting the broad impact of *Salmonella* on medicine, veterinary medicine, and public health. While antibiotics have traditionally been effective in preventing and controlling *Salmonella*, the rise of antibiotic-resistant strains poses a significant challenge (Castro-Vargas et al., 2020). This emergence of drug-resistant and even super-resistant bacteria complicates *Salmonella* prevention and control efforts (Cuypers et al., 2018; Castro-Vargas et al., 2020), posing threats to both the poultry industry development and public health safety.

*Salmonella* is a prevalent pathogen linked to foodborne illness on a global scale, as noted by the World Health Organization (2016b). Outbreaks of these illnesses are often linked to the consumption of eggs, chicken, raw meat, and contaminated water (Ran et al., 2011). Hence, swift and accurate detection methods are essential for monitoring food quality and eliminating pathogens in chicken farms, ultimately safeguarding consumer health and fostering advancements in the poultry industry. Isolation and culture are the traditional methods for *Salmonella* detection, according to ISO 6579:2002 (International Organization for Standardization, 2007), with the disadvantage of being time- and labor-intensive. Biosensors are preferred over traditional detection methods due to their heightened sensitivity and speed (Bazin et al., 2017). Bacteriophages, which are bacterial viruses, exhibit remarkable specificity and can differentiate between living and dead bacteria. Consequently, the utilization of engineered bacteriophages in the detection of *salmonella* is gaining increasing interest (Fernandes et al., 2014).

Bacteriophages, as a type of virus that can specifically recognize and lyse bacteria (O’Flaherty et al., 2009), have garnered increasing attention from scientific researchers due to the growing concern over bacterial drug resistance (Strathdee et al., 2023). With advancements in biotechnology and a deeper understanding of bacteriophage research, bacteriophage therapy is poised to enter a new era of significance (Kingwell, 2015; Strathdee et al., 2023). Bacteriophages, as novel antibacterial agents, have been extensively researched and are now being approved for various applications in biotechnology (Islam et al., 2020; Hatfull et al., 2022). Despite the perceived limitation of high specificity hindering further development (Zalewska-Piątek, 2023) and utilization of bacteriophages, this characteristic can be leveraged for the targeted detection of specific microorganisms (Drulis-Kawa et al., 2015). The unparalleled sensitivity and specificity of bacteriophages (Singh et al., 2010) in organic systems surpasses that of inorganic reagents. The foundation for this high specificity lies in the receptor binding protein (RBP) of each bacteriophage. Tail fiber proteins, as a type of bacteriophage RBP (Nobrega et al., 2018), play a crucial role in the specific recognition of host bacteria (Taslem Mourosi et al., 2022). For host bacteria, the bacteriophage receptors on their surface serve as essential sites that must be identified and engaged during the process of bacteriophage infection (Nobrega et al., 2018; Klumpp et al., 2023).

This study utilized protein spectrometry to identify the functions of multiple proteins of the SP bacteriophage YSP2, which was isolated in previous work (Tie et al., 2018), particularly focusing on the tail fiber protein 35Q. The prokaryotic expression system was employed to induce the expression of this protein. Subsequently, the adsorption activity of tail fiber protein 35Q on the bacterial surface was examined. This research aimed to determine the receptors of bacteriophage YSP2 on the bacterial surface and its character, providing a theoretical foundation for the potential application of bacteriophage YSP2 and its derivatives in *Salmonella* detection and specific elimination in chicken farms.

## 2 Materials and methods

### 2.1 Bacteriophage, bacterial strains, and culture conditions

Details of bacterial strains used in the study are listed in Table 1. All bacterial strains were stored at −80°C and routinely grown at 37°C. Clostridium perfringens was cultured in brain heart infusion (BHI) broth, while others were cultured in Luria-Bertani (LB) medium. According to the multiplicity of infection (MOI) of YSP2, added bacteriophage to the culture of SP until the culture was clear, then the culture was filtered through Millipore filters (0.22-μm pore size) to obtain the YSP2 suspension.

**Table 1:** Source of bacteriophage and bacterial strains.

Organism	Strain	Source
*Salmonella* pullorum	SP	1
*Salmonella* bacteriophage	YSP2	1
*Escherichia coli*	DH5α	2
*Escherichia coli*	BL21	2
*Escherichia coli*	K88	3
*E. coli* Bacteriophage	KP	4
*C. perfringens*	ATCC13124	5
*C. perfringens* Bacteriophage	AP	4

1, isolated from the farm; 2, purchased from TIANGEN Biotech (Beijing) Co., Ltd; 3, purchased from China Institute of Veterinary Drug Control; 4, isolated from various sewerage systems in Changchun; 5, purchased from the American Type Culture Collection.

### 2.2 Bacteriophage concentration and purification

High concentrations of bacteriophage were obtained using PEG precipitation methods (Sweere et al., 2019), for DNA extraction, and to purify bacteriophage using cesium chloride density gradient centrifugation. To prepare the CsCl solution as shown in Table 2, the sample was centrifuged horizontally at 35,000 rpm for 3 h. Carefully the purified light blue bacteriophage suspension (Supplementary Figure S1) was collected for Q Exactive (Shanghai Applied Protein Technology Co., Ltd. More detail has been added Supplementary material).

**Table 2:** Preparation of CsCl gradient solution.

Density (g/mL)	CsCl (g)	SM buffer (mL)
1.32	4.20	9.00
1.45	6.00	8.50
1.50	6.70	8.20
1.70	9.50	7.50

### 2.3 Amino acid sequence analysis of putative tail fiber protein

Early work sequenced the whole genome of bacteriophage YSP2 (Tie et al., 2018), in which ORF35 is a sequence putatively encoding a tail fiber protein. The amino acid sequence (YP_009796010.1) was aligned using BLAST,^1^ and several phylogenetically related protein amino acid sequences were used to reconstruct a phylogenetic tree using MEGA7 software (Felsenstein, 1985; Saitou and Nei, 1987; Kumar et al., 2016). After analysis using NCBI’s analysis tools, a 1,503 bp sequence *35q* encoding the tail fiber protein was selected.

### 2.4 Cloning, expression, and purification of bacteriophage tail fiber protein

A 1503-bp DNA fragment containing the 35q gene was amplified by a polymerase chain reaction (PCR) using bacteriophage YSP2 genomic DNA as a template and primers 35QF (CGGGATCCATGGCTTTATATCGCACGGGCACGG) and 35QR (CCGCTCGAGTTACCATGCACCCCAAGAGCCATCA). Restriction endonuclease sites for *BamH I* and *Xho I* were included at the 5’ends of 35QF and 35QR, respectively. Primers were synthesized by Shanghai Shengong Bioengineering Co., Ltd., Shanghai, China. The PCR product was digested with *BamH I* (NEB) and *Xho I* (NEB) and ligated into the corresponding sites of vector pET-28a (+) using T4 DNA ligase (Takara) to construct pET-28a-35Q. The ligation product was transformed into *Escherichia coli* (*E. coli*) strain DH5α, and positive transformants were selected using the Kan + (50 μg/mL) low salt LB solid medium. The full expression vector, pET-28a-35Q, was transformed into *E. coli* BL21 competent cells. Exponentially growing cultures were induced with 1 mM Isopropyl β-D-Thiogalactoside (IPTG). Bacterial cells were washed with phosphate-buffered saline (PBS), disrupted with an ultrasonic disintegrator, and centrifuged at 4°C (10,000 × g, 15 min). The supernatant and centrifuge pellet were analyzed by SDS-PAGE using 12% gels. For protein purification, the supernatant was dialyzed against PBS, added to Ni-nitrilotriacetic acid (NTA) (nickel matrix) His-Bind slurry, and eluted according to the manufacturer’s instructions (Merck-Novagen).

### 2.5 Verify the adsorption activity and specificity of tail fiber protein 35Q

To evaluate 35Q adsorption to SP, tail fiber protein was added to the SP bacterial suspension. After a ten-minute incubation at 37°C, according to the MOI (Tie et al., 2018), YSP2 was added, mixed thoroughly, and incubated at 37°C for 10 min. Subsequently, centrifugation at 10,000 g for 15 min at 4°C was carried out. The collected supernatant underwent quantification of free YSP2 concentration using the double-layer agar method (*n* = 3) (Lindberg et al., 1978; Parent et al., 2014). A mixture of SP and YSP2 was prepared as a negative control, while a mixture of SM buffer and YSP2 was prepared as a blank control. Subsequently, the supernatants were subjected to centrifugation under identical conditions to count the free bacteriophage (*n* = 3).

To examine the adsorption specificity of tail fiber protein 35Q, the non-target bacteria of *E. coli*, *Clostridium perfringens* and corresponding bacteriophages KP (*E. coli*), AP (*Clostridium perfringens*) were tested simultaneously (*n* = 3).

### 2.6 Identification of receptors bound by tail fiber protein 35Q on the bacterial surface

To identify the receptors with which tail fiber protein 35Q binds on the bacterial surface, ELISA experiments were used to verify the binding of tail fiber protein to bacterial outer membrane protein (**OMP**) and lipopolysaccharide (**LPS**). SP was grown overnight in liquid media and the bacteria were pelleted by centrifugation. After washing with PBS, the kit was used to extract OMP and LPS of SP, respectively.

High-binding microtiter plates were coated overnight at 4°C with OMP (10 mg/mL) diluted 1/10 in coating buffer (carbonate–bicarbonate buffer, pH 9.6). Poured off the coating buffer, washed the reaction wells with 200 μL wash buffer (PBST) 6 times, added 100 μL blocking buffer (2% BSA) to each well, and blocked at 37°C for 2–3 h. After washing 6 times, added purified 35Q (400 μg/mL), in parallel experimentation, Tris-NaCl was added to other wells as a negative control, and incubated at 37°C for 2 h. Added Tris-NaCl to the blank coating buffer and performed the same treatment as the blank group. Added 50 μL of His-tag antibody diluted at 1:3000 to all wells, react at 37°C for 1 h, and wash 6 times; added 50 μL of HRP-conjugated secondary antibody at 1:5000 react at 37°C for 1 h to amplify the signal, after washing 6 times, developed the colorimetric signal with TMB substrate solution, incubate at 37°C for 15–30 min in the dark, stop the reaction with stop buffer (2 M sulfuric acid) and measured absorbance at 450 nm using a microplate reader (n = 3).

To make the binding strength between LPS and microtiter plates stronger (Morrison and Jacobs, 1976), dissolved Polymyxin B (PMB) in the coating buffer (10 mg/mL), added 100 μL to the well for pre-coating, and incubated at 4°C overnight. The subsequent ELISA operation steps are the same as those of OMP (*n* = 3).

### 2.7 Adsorption assay to verify bacteriophage binding sites

In order to confirm the nature of the bacteriophage binding site on the bacterial surface, proteinase K and potassium periodate were used to destroy OMP and LPS on the bacterial surface respectively, and then bacteriophage YSP2 was used to perform an adsorption test, and the binding site was preliminarily determined based on the concentration of free bacteriophage in the supernatant.

SP cultured overnight was washed with PBS and resuspended in an equal volume of 0.2 mg/mL proteinase K, incubated at 37°C for 2–3 h, and washed and resuspended in an equal volume of PBS. Bacteria were treated similarly with PBS as a negative control. Added 20 μL of bacteriophage YSP2 to them, incubated at 37°C for 10 min, immediately centrifuged at 4°C, took the supernatant, and used the double-layer agar method to calculate the concentration of free YSP2 in the supernatant (*n* = 3), an equal amount of bacteriophage YSP2 was added to SM buffer for the same treatment as a blank control (*n* = 3).

After using 0.02% KIO_4_ to destroy the LPS of SP (Steine et al., 2001), performed the same adsorption test as above (*n* = 3).

### 2.8 Statistical analysis

The data were expressed as mean ± SD. All experiments were independently performed three times. *t*-tests were applied to assess the difference when two groups were compared. Statistical analysis was performed using GraphPad Software (GraphPad Software Inc., Version 6.01 San Diego, CA, United States).

## 3 Results

### 3.1 Analysis of global amino acids of bacteriophage YSP2

The global protein of bacteriophage YSP2 was analyzed by mass spectrometry using Q Exactive and compared with a known bacteriophage protein library. Mass spectrometry analyzed a total of 52 protein amino acid sequences of bacteriophage YSP2, many of which had predicted functions based on comparisons in the database: Tail fiber protein, Capsid protein, Single-strand DNA binding protein, Coil containing protein, DNA primase/polymerase Lyase, Endopeptidase, etc. (More in Supplementary Table S1 and Supplementary Figure S2). Due to the specificity of YSP2 and the broad spectrum of its endolysin LySP2 (Deng et al., 2023), the tail fiber protein of YSP2 was selected as the main research object in this study.

### 3.2 Comparative analysis of amino acids encoded by ORF35

**Figure 1:**
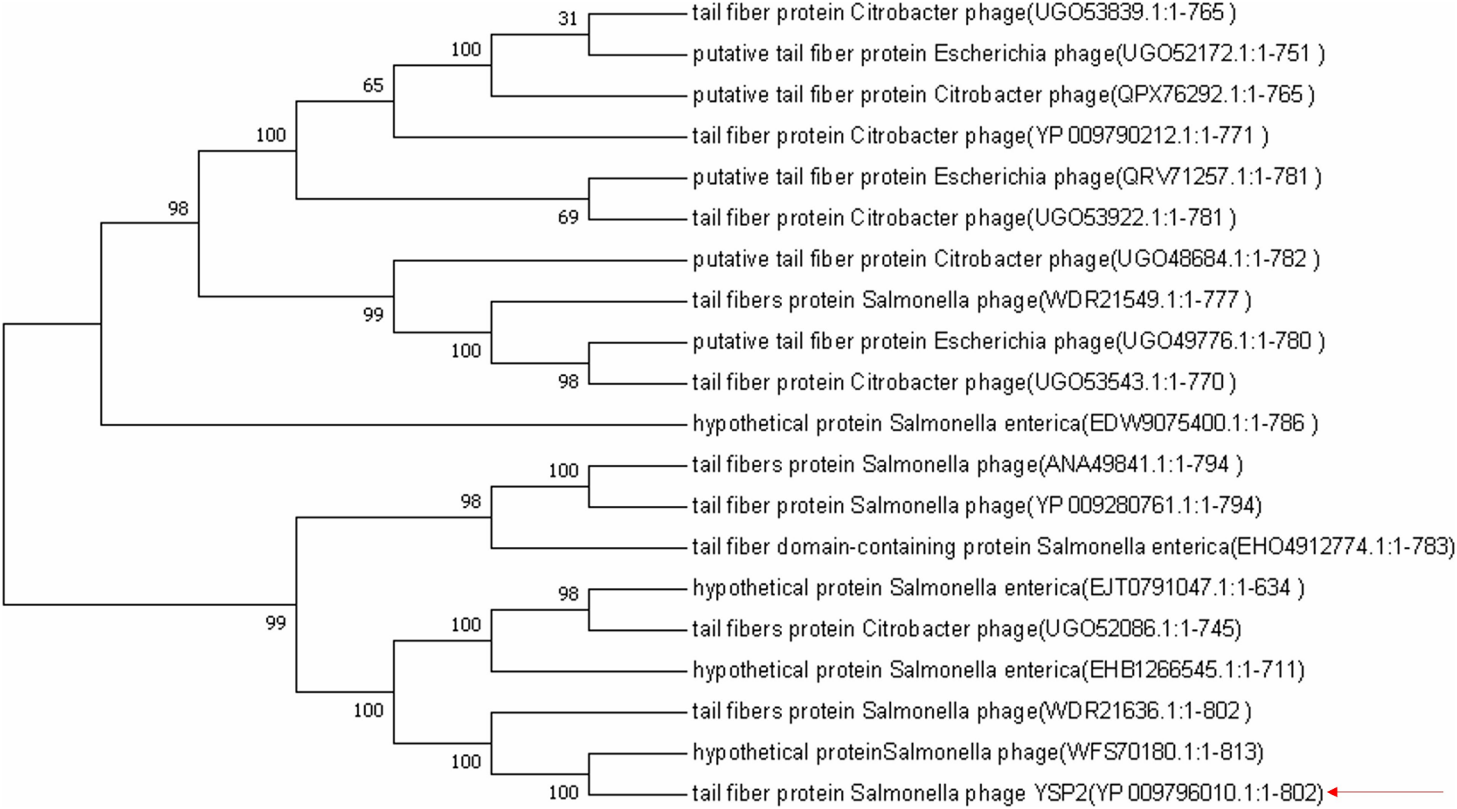
Neighbor-joining phylogenetic tree based on the amino acid fragment of 35Q and related sequences. Bootstrap values >50% (based on 1,000 replicates) are shown at branch points. GenBank accession numbers are given in parentheses following bacteriophage name.

ORF35 encodes 802 amino acids, and BLASTX analysis showed that the amino acid sequence is highly similar to the amino acids of tail fiber protein from other *Salmonella* bacteriophages. The amino acid sequence used for the BLAST analysis was employed for phylogenetic tree construction, which showed that tail fiber protein amino acids from other bacteriophages are closely related to that from YSP2 (Figure 1). After analyzing the amino acid function of ORF35 and comparing the data in the database, a truncated fragment *35q* with a length of 1,503 bp was selected (Supplementary Figure S3). The homology of 35Q with *Salmonella* bacteriophage phaSE-2 tail fiber protein, *Salmonella* bacteriophage vB_SenS_PHB07 tail fiber protein and Citrobacter bacteriophage CF1 DK-2017 tail fiber protein is 83.40, 61.20 and 60.40%, respectively (Supplementary Figure S4).

### 3.3 Construction and expression of 35Q

**Figure 2:**
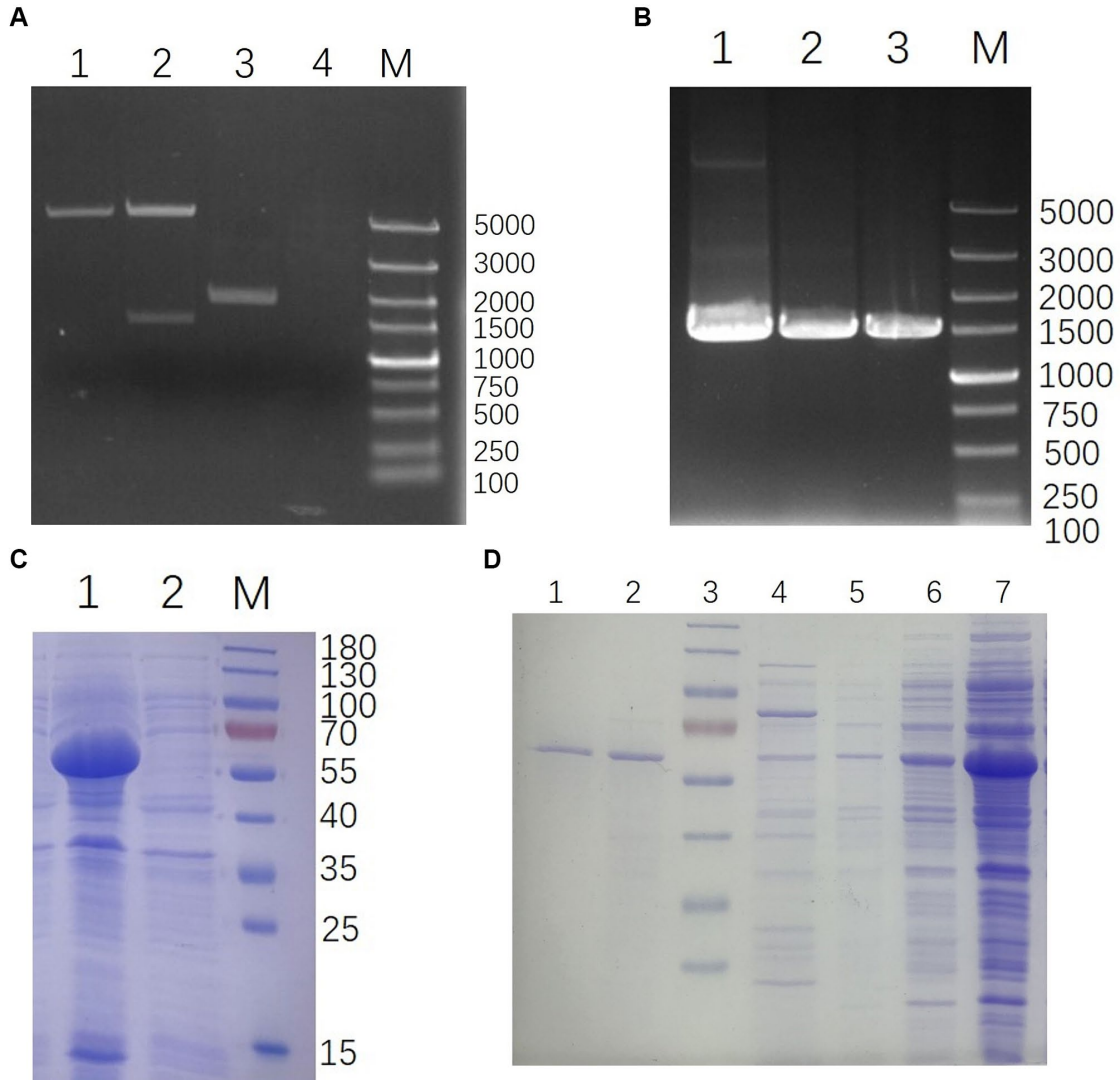
Construction and expression of 35Q. **(A)** Verification of pET-28a-35Q by enzyme digestion. Lanes: 1, pET-28a digestion with *BamH I* and *Xho I*; 2, pET-28a-35Q digestion with *BamH I* and *Xho I*; 3, Recombinant plasmid pET-28a-35Q; 4, H_2_O; M, DL 5,000 DNA marker. **(B)** Identification of recombinant expression strain BL21-pET-28a-35Q by PCR. Lanes: 1, BL21-pET-28a-35Q PCR amplification product; 2, pET-28a-35Q PCR amplification product; 3, *35q* PCR amplification product; M, DL 5,000 DNA marker. **(C)** Induced products analyzed by SDS-PAGE. Lanes: 1, Induced products of BL21-pET-28a-35Q by IPTG; 2, Uninduced strain BL21-pET-28a-35Q; M, 180 kDa protein ladder. **(D)** Purification of 35Q. Lanes: 1–2, the purified 35Q fraction eluted from Ni-NTA His-Bind slurry; 3, 180 kDa protein ladder; 4–5, Imidazole eluent; 6–7, Supernatant of the induced BL21-pET-28a-35Q after being crushed.

The whole genome of YSP2 (GenBank: MG241338.1) was extracted and used as a template to amplify the target gene 35Q by PCR and used to construct the recombinant plasmid pET-28a-35Q. After verification by double enzyme digestion (Figure 2A) and PCR (Figure 2B), the recombinant plasmid pET-28a-35Q was transformed into competent BL21 cells to obtain the recombinant expression strain BL21-pET-28a-35Q. Exponentially growing cultures of *E. coli* BL21-pET-28a-35Q were induced with 1 mM IPTG, followed by incubation for 12 h at 16°C with shaking at 120 rpm. As seen in (Figure 2C), SDS-PAGE revealed that there was a band of approximately 60 kDa, corresponding to the predicted size of the bacteriophage tail fiber protein 35Q. This band was absent from uninduced cells. In addition, 35Q was found in the supernatant as a fusion protein. A homogeneous band also emerged in a sample of purified His-tagged bacteriophage tail fiber protein (Figure 2D).

### 3.4 Adsorption activity and specificity of tail fiber protein 35Q

**Figure 3:**
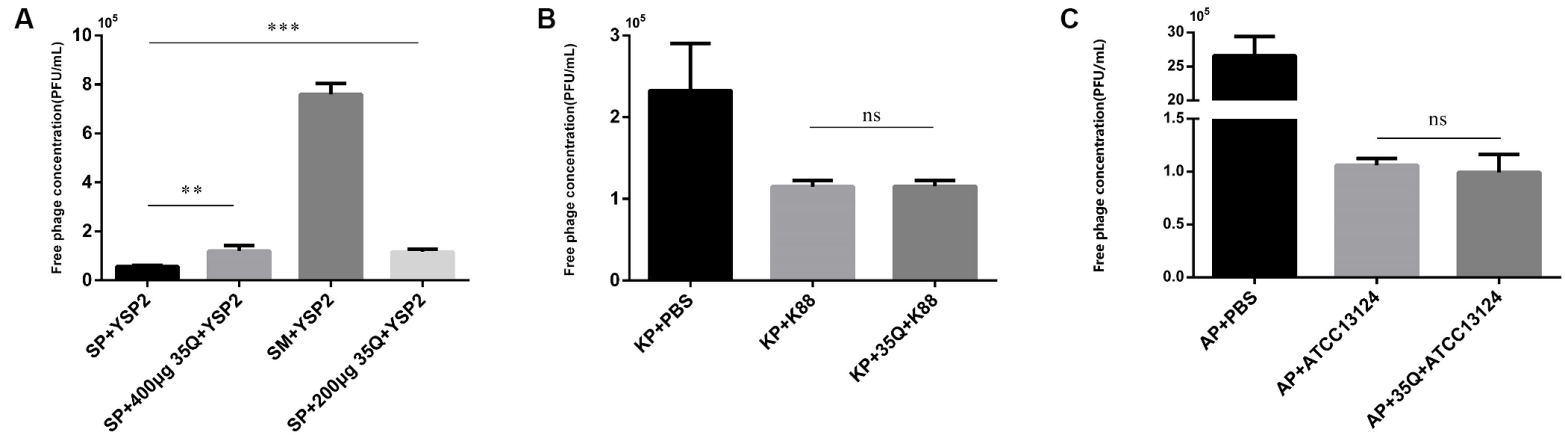
The adsorption activity and specificity of 35Q. **(A)** Tail fiber protein 35Q competes with *Salmonella* bacteriophage YSP2 to adsorb receptors, and the concentration of free bacteriophages reflects adsorption activity (*n* = 3). **(B)** 35Q competes with *E. coli* bacteriophage KP to adsorb receptors (*n* = 3). **(C)** 35Q competes with *Clostridium perfringens* bacteriophage AP to adsorb receptors (*n* = 3) (^*^p < 0.05, ^**^p < 0.01, ^***^p < 0.001 and “ns” means not significant).

The tail fiber protein 35Q competes with the bacteriophage YSP2 for binding sites on the bacterial surface, and the adsorption activity of the protein is reflected by the concentration of the free bacteriophage. As shown in Figure 3A, there was a difference between group SP + YSP2 and group SP + 400 μg 35Q + YSP2 (*p* < 0.001), and it’s same between SP + YSP2 and group SP + 200 μg + YSP2 (*p* < 0.001), which showed that protein 35Q adsorbate receptors on the surface of SP and formed competitive adsorption with bacteriophage, indicating that the tail fiber protein 35Q had adsorption activity.

The specificity of tail fiber protein 35Q was determined. In Figure 3B, *E. coli* k88 was incubated with tail fiber protein 35Q and then subjected to adsorption tests with the corresponding bacteriophage KP. Compared with the adsorption test in the control group, the concentration of free bacteriophage in the supernatant did not increase, and there was no statistical difference between the two groups (*p* = 0.958). Similarly, experiments on *Clostridium perfringens* shown in Figure 3C yielded similar result (*p* = 0.560). The above results showed that the tail fiber protein 35Q only adsorbed SP and did not adsorb non-targeted strains, revealing the adsorption specificity of 35Q to *Salmonella*.

### 3.5 Identification of the binding targets of tail fiber protein 35Q

**Figure 4:**
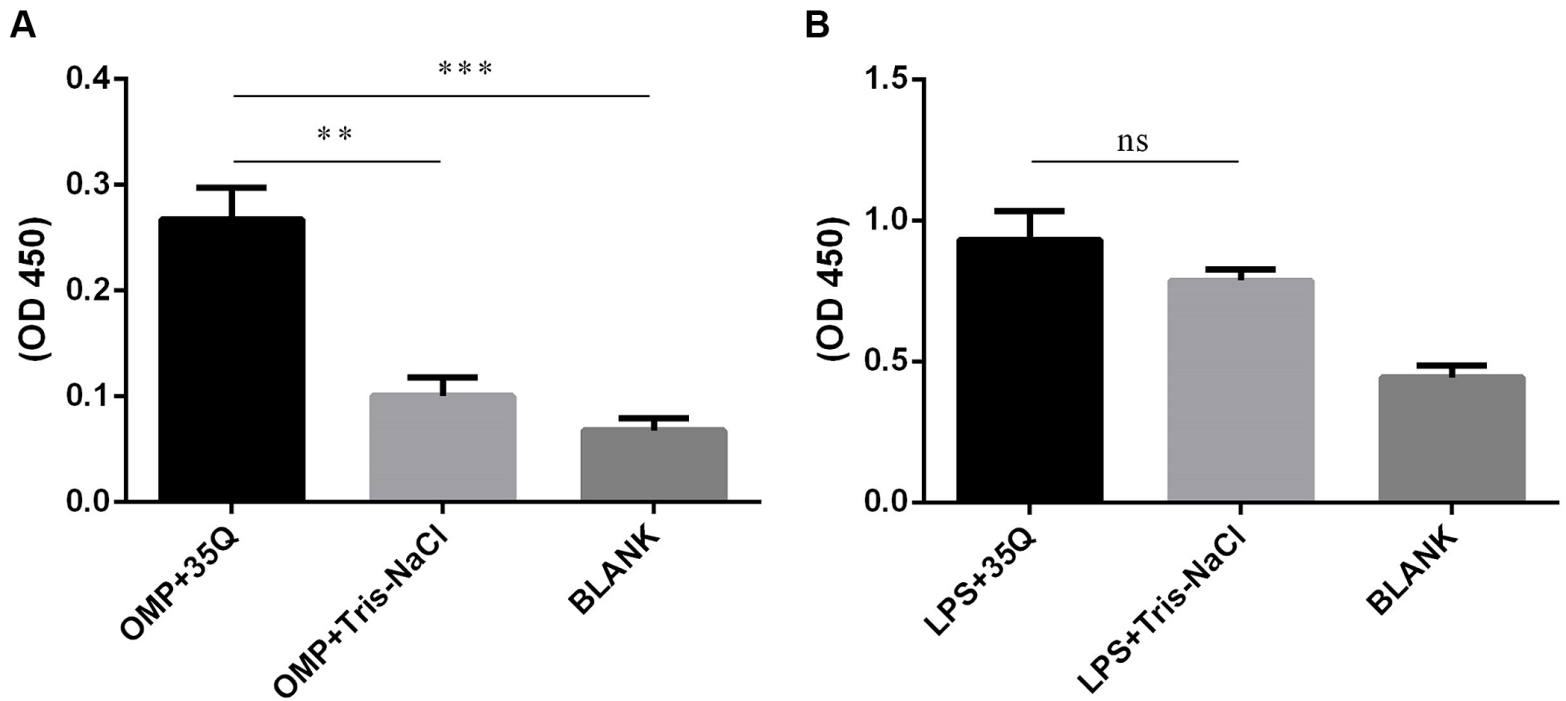
Identification of the binding targets of tail fiber protein 35Q. **(A)** ELISA results of 35Q and OMP of SP (*n* = 3). **(B)** ELISA results of 35Q and LPS of SP (*n* = 3) (^*^p < 0.05, ^**^p < 0.01, ^***^p < 0.001 and “ns” means not significant).

The results of ELISA were obtained by combining the OMP and LPS of *Salmonella* with tail fiber protein 35Q. As shown in Figure 4A, the OD450 intensity of the “OMP + 35Q” group was significantly higher than that of the negative control group, and the difference was significant. ELISA experiments to verify the binding of LPS and 35Q showed that there was no difference between the experimental group and the negative control group as shown in Figure 4B.

### 3.6 Characterization of bacteriophage binding sites on bacterial surface

**Figure 5:**
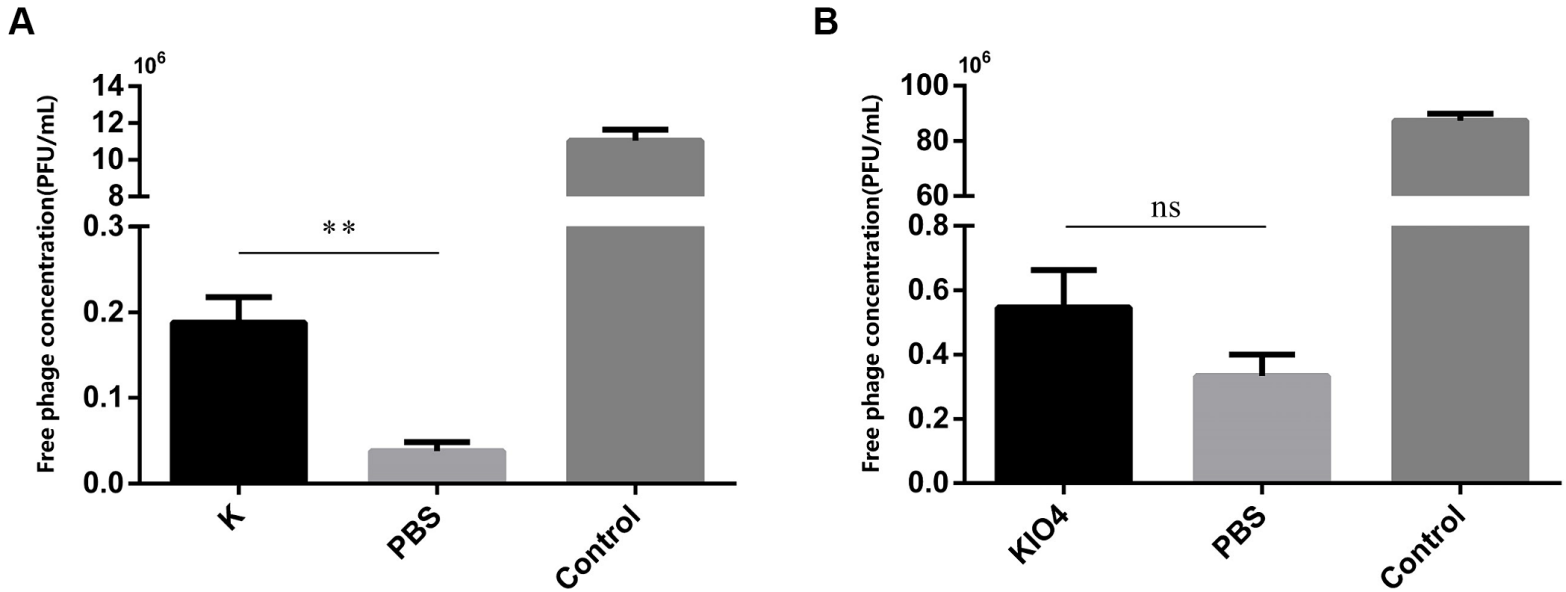
Characterization of bacteriophage binding sites on bacterial surface. **(A)** Adsorption test of SP treated with protease K and bacteriophage YSP2 (K: SP treated with protease K; PBS: SP treated with PBS; Control: YSP2 diluted with an equal amount of SM buffer; *n* = 3). **(B)** Adsorption test of SP treated with KIO_4_ and bacteriophage YSP2 (KIO_4_: SP treated with KIO_4_; PBS: SP treated with PBS; Control: YSP2 diluted with an equal amount of SM buffer; *n* = 3) (^*^p < 0.05, ^**^p < 0.01, ^***^p < 0.001 and “ns” means not significant).

After treatment with proteinase K, the surface protein of SP was degraded. If OMP is the receptor for bacteriophage adsorption, then the bacteriophage adsorption rate will be reduced after treating the host with proteinase K. As shown in Figure 5A, compared with the PBS-treated group, the bacteriophage concentration in the supernatant after centrifugation in the proteinase K-treated group was greatly increased, there was a difference (*p* = 0.001). The bacteriophage adsorption rate dropped significantly. This result shows that the nature of the receptor adsorbed by bacteriophage YSP2 during the infection of bacteria is the OMP on the surface of SP. KIO4 can oxidatively break the ortho-dihydroxyl groups of sugar molecules to generate the corresponding polysaccharide aldehyde, formaldehyde or formic acid, which changes the structure of LPS. Similarly, if SP bacteriophage YSP2 binds to LPS on the bacterial surface, the bacteriophage adsorption rate decreases after the host is treated with KIO4. As shown in Figure 5B, compared with the PBS-treated group, the bacteriophage concentration in the supernatant after centrifugation in the KIO4-treated group increased slightly, but the difference was not significant and there was no statistical difference (*p* = 0.0506). The adsorption rate of bacteriophage did not decrease significantly. This result shows that LPS is not the main receptor for bacteriophage adsorption and binding.

## 4 Discussion

Since the discovery of bacteriophages (Letarov, 2020), much attention has been given to their role in bactericidal activity (Cisek et al., 2017). Nevertheless, their uncomplicated structure and diverse protein functions have significantly broadened our comprehension of biological diversity (Marks and Sharp, 2000). The study of bacteriophage functions and life processes has yielded valuable reagents for molecular biology research, such as restriction endonucleases and polymerases (Pingoud et al., 2014; Safari et al., 2020). Furthermore, the mutual adaptation and co-evolutionary relationship between bacteriophages and bacteria are crucial for understanding global ecosystems and evolution (Safari et al., 2020; Shen J. et al., 2023).

Bacteriophages have emerged as a promising solution in combating drug-resistant bacteria (Lin et al., 2017), with some even being successfully utilized in clinical settings (Tan et al., 2021; Dedrick et al., 2023). They can be employed for targeted bacterial detection due to their specificity towards host bacteria (Wang et al., 2017; He et al., 2018; Liu et al., 2022). Additionally, biotechnological advancements have enabled the expression of bacteriophage lytic enzymes and endolysins, expanding the spectrum of bacteriophage lysis and facilitating the development of novel antibacterial treatments (Schuch et al., 2019; Deng et al., 2023; Shen K. et al., 2023).

Bacteriophage YSP2, a highly lytic bacteriophage previously isolated in our research (Tie et al., 2018), demonstrates a specific lysis spectrum targeting *Salmonella*. Interestingly, the endolysin Lysp2 derived from bacteriophage YSP2 exhibits the ability to lyse both *Salmonella* and *E. coli* (Deng et al., 2023), while the complete bacteriophage YSP2 is unable to lyse the test bacteria ATCC25922. The intriguing phenomenon has sparked our interest in the tail proteins responsible for bacteriophage specificity. Our research is centered on identifying tail fiber protein and exploring its potential future applications. Whole protein mass spectrometry analysis and whole genome sequencing were performed on bacteriophage YSP2, leading to the identification of the tail fiber protein 35Q. Amino acid analysis of 35Q revealed that the majority of its homologous proteins were bacteriophage tail fiber proteins or putative tail fiber protein, with many originating from *Salmonella* bacteriophages. This finding helps to explain why YSP2 exhibits strong specificity in cleaving multiple strains of *Salmonella*. The expression and purification of tail fiber protein 35Q not only provide a material basis for the specific detection of *Salmonella*, but also offer potential for enhancing food safety and pathogen elimination in livestock and poultry farms. The specific recognition and adsorption characteristics of this protein have the potential to significantly improve detection accuracy, making it a promising area for future research and application.

Tail fiber protein 35Q was expressed using a prokaryotic expression system, and after purification and concentration, the concentration reached 4 mg/mL. In the experiment to verify protein activity, the adsorption activity of the tail fiber protein was measured under MOI conditions by competing with bacteriophages to adsorb bacterial surface receptors. The concentration of free bacteriophages in the supernatant was measured to reflect the adsorption activity of 35Q. In the adsorption experiment, 200 μg and 400 μg tail fiber protein were intentionally used, but no statistical difference was found between the two groups (*p* = 0.829). This suggests that under the given conditions, 200 μg tail fiber protein was adequate to reach the required concentration, enhancing the reliability of the adsorption experiment. Following pre-treatment with tail fiber protein 35Q, the adsorption rate of bacteriophage YSP2 on *Salmonella* showed a significant decrease compared to the control group. Conversely, when non-targeted bacteria like *E. coli* (Gram-negative) and *Clostridium perfringens* (Gram-positive) were treated with 35Q, their corresponding bacteriophage adsorption rates remained unaffected, highlighting the high specificity of 35Q. High concentration, high adsorption activity, and high specificity also provide a guarantee for subsequent experiments and future applications. As for future applications and prospects, after obtaining such a specific binding protein, our subsequent experiments can conduct more in-depth research in the areas of specific detection of bacteria, bacterial localization imaging, and drug-carrying enhanced targeted delivery.

The specific binding mechanism between bacteriophages and bacteria plays a crucial role in targeting receptors on the surface of bacteria and ultimately killing them. Components such as the tail protein of bacteriophages, LPS and OMP on the surface of bacteria, serve as the foundation for this specific binding. Bacteriophages identify their host bacteria by attaching to particular surface receptors, such as OMP, LPS, or elements of the bacterial capsule, pili, and flagella (Rakhuba et al., 2010; Bertozzi Silva et al., 2016; Letarov and Kulikov, 2017). Numerous studies have shown that the primary bacteriophage receptors for gram-negative bacteria are LPS and OMP located on the bacterial surface (Munsch-Alatossava and Alatossava, 2013; Van Den Berg et al., 2022; Degroux et al., 2023). In this study, we selected OMP and LPS as pseudo receptors to further validate the binding receptors of bacteriophage YSP2 on the surface of *Salmonella*. We conducted ELISA experiments to confirm the binding of tail fiber protein 35Q with OMP and LPS of *Salmonella*. The results showed that tail fiber protein 35Q specifically bound to the OMP on the surface of *Salmonella*.

In order to validate our findings, we conducted an adsorption test of bacteriophage YSP2 on SP with the destruction of OMP and LPS. We observed the adsorption of intact bacteriophages and treated bacteria. The results indicated that upon the destruction of OMP by protease K, there was a notable increase in the concentration of bacteriophages in the supernatant solution, the bacteriophage adsorption rate decreased significantly, showing a significant difference from the control group (*p* < 0.01). In the adsorption test of bacteriophages and *Salmonella* that destroyed LPS, the concentration of free bacteriophages in the solution did not significantly increase, and the adsorption rate of bacteriophages did not decrease significantly compared to the control group (*p* = 0.0506). Although the observed difference is not statistically significant, the *p*-value is close to the critical threshold (*p* = 0.05). This could be attributed to the destruction of other potential receptors or non-primary receptors on the surface of SP after KIO_4_ treatment, resulting in a slight decrease in the adsorption rate of bacteriophages. Since there are multiple types of receptors for bacteriophages on the surface of bacteria, further in-depth research is necessary to understand the interaction mechanism between SP and bacteriophage YSP2.

A substantial number of experiments are required to confirm the molecular mechanisms underlying the interaction between bacteriophage RBP and various receptors on the bacterial surface. For example, interacting proteins are separated through pull-down experiments and the corresponding amino acid sequences are obtained through sequencing mass spectrometry. More accurate protein interaction verification methods can be used to verify the interaction between RBP and receptor, such as bimolecular fluorescence complementation technology. The mechanism of interaction is then elucidated using other methods such as structure prediction and point mutation. On the other hand, gene editing can be used to knock out or silence the coding genes of the host bacterial surface structure to study the effect on bacteriophage adsorption. Furthermore, understanding how to leverage the specificity of bacteriophages for detecting specific pathogens, engineering bacteriophages to enhance their lysis spectrum, and utilizing bacteriophages and their derivatives in conjunction with antibiotics for bacteriophage therapy play crucial roles in reducing and limiting antibiotic usage.

## Data availability statement

The original contributions presented in the study are included in the article/Supplementary material, further inquiries can be directed to the corresponding authors.

## Author contributions

HD: Writing – original draft, Writing – review & editing. LF: Validation, Writing – review & editing. KS: Conceptualization, Writing – review & editing. RD: Conceptualization, Writing – review & editing.

## Footnotes

^1^https://blast.ncbi.nlm.nih.gov
