# Supplementary material for: Binding activity and specificity of tail fiber protein 35Q for Salmonella pullorum
Source: Front Microbiol. 2024 Jun 25;15:1429504. doi: 10.3389/fmicb.2024.1429504 (PMC11231377; doi:10.3389/fmicb.2024.1429504)
Supplement: Supplementary file 1 [file Data_Sheet_1.PDF]

Supplementary Material

Q Exactive Identification Analysis of Bacteriophage YSP2

YSP2 enzymatic hydrolysis:

(1) Mix 30μL YSP2 with DTT to a final concentration of 100mM DTT. Incubate at 95°C for 5 min and cool to room temperature.

(2) Add 200μL UA buffer (8M Urea, 150mM Tris-HCl pH8.5), mix, and transfer to a 10kDa centrifugal filter unit. Centrifuge at 14000g for 30 minutes and discard the flow-through.

(3) Add 200μL UA buffer (8M Urea, 150mM Tris-HCl pH8.5) to the filter unit and mix. Centrifuge at 14000g for 30 minutes and discard the flow-through.

(4) Repeat step (3).

(5) Add 100 μ L IAA（100mM IAA in UA）to the filter unit and incubate at room temperature in dark for 30 minutes. Centrifuge at 14000g for 30 minutes and discard the flow-through.

(6) Add 100μL UA buffer (8M Urea, 150mM Tris-HCl pH8.5) to the filter unit and mix. Centrifuge at 14000g for 30 minutes and discard the flow-through.

(7) Repeat step (6).

(8) Add 100μL 2mM NH_4HCO_3 to the filter unit and mix. Centrifuge at 14000g for 30 minutes. and discard the flow-through.

(9) Repeat step (8).

(10) Add 48 μ L Trypsin buffer（4 μ g Trypsin in 40 μ L 25mM NH_4HCO_3）to the filter unit and spin at 600rpm for 1 minute. Incubate at 37℃ for 16-18 hours.

(11) Centrifuge at 14000g for 30 minutes and collect the flow through.

(12) Add 40 μ L 25mM NH_4HCO_3， centrifuge at 14000g for 30 minutes.

(13) Collect the flow through and determine the concentration of trypsin-digested peptides by UV spectrometry at 280 nm.

High-Performance Liquid Chromatography (HPLC)

Separating peptides of a sample by using Easy nLC system (Thermo Scientific). The Peptides were loaded in 95% solvent A (0.1% formic acid) on a two-column set-up consisting of a trap column（Thermo Scientific Acclaim PepMap100, 100μm*2cm, nano Viper C18）and an analytical column（Thermo scientific EASY column, 15cm, ID150μm, 3μm, C18）.

Mass Spectrometry Assay

Eluted peptides were analyzed using the Q-Exactive mass spectrometer (Thermo Scientific) for 60 min. The mass spectrometer was operated in positive ion mode. Full scan MS spectra (m/z 300–1800) were acquired with a resolution of 70000 at 100 m/z. Automatic gain control (AGC) target was set to 1e6; Maximum inject time was 50 ms, and dynamic exclusion was 30.0s. Up to 20 most intense ions were selected for higher-energy collisional dissociation fragmentation depending on signal intensity. Isolation window was1.5m/z; MS/MS fragment spectra were acquired with a resolution of 17500 at 100 m/z， AGC target was 1e5; Maximum inject time was 50 ms; Normalized collision energy was 27eV and underfill was0.1%.

Mass Spectrometry Assay

The raw file of the mass spectrometry test was searched with Mascot2.2 software to find the corresponding database, and finally, the identified protein results were obtained.

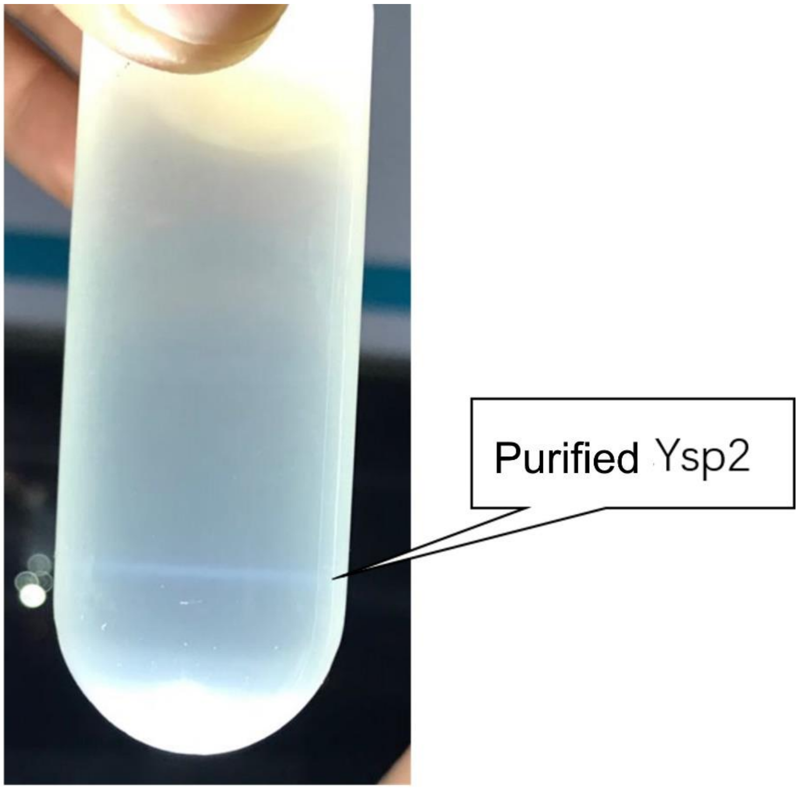

Fig.S1 Bacteriophage YSP2 after density gradient centrifugation

Table S1 Phage YSP2 protein mass spectrometry identification

	Pep Count	Score	MW(KDa)	Annotated function
1	5	29.3	45.2	Tail fiber protein
2	7	30.5	43.8	Capsid protein
3	5	31.3	24.8	Single-strand DNA binding protein
4	8	64.3	28.4	Coil containing protein
5	1	47.3	54.2	Chaperonin
6	1	20.9	44.5	DNA primase/polymerase
7	17	149.7	22.9	NinG protein
8	1	47.6	63.5	Portal protein
9	5	55.5	143	Tape measure protein
10	1	20.5	34	Terminase
11	1	26.5	34.6	Replication initiation protein
12	2	21.9	28.9	Lyase
13	2	30.5	145.3	Endopeptidase
14	3	110.7	108.2	Endo-N-acetylneuraminidase
15	1	30	10.4	Galactose-1-phosphate uridylyltransferase

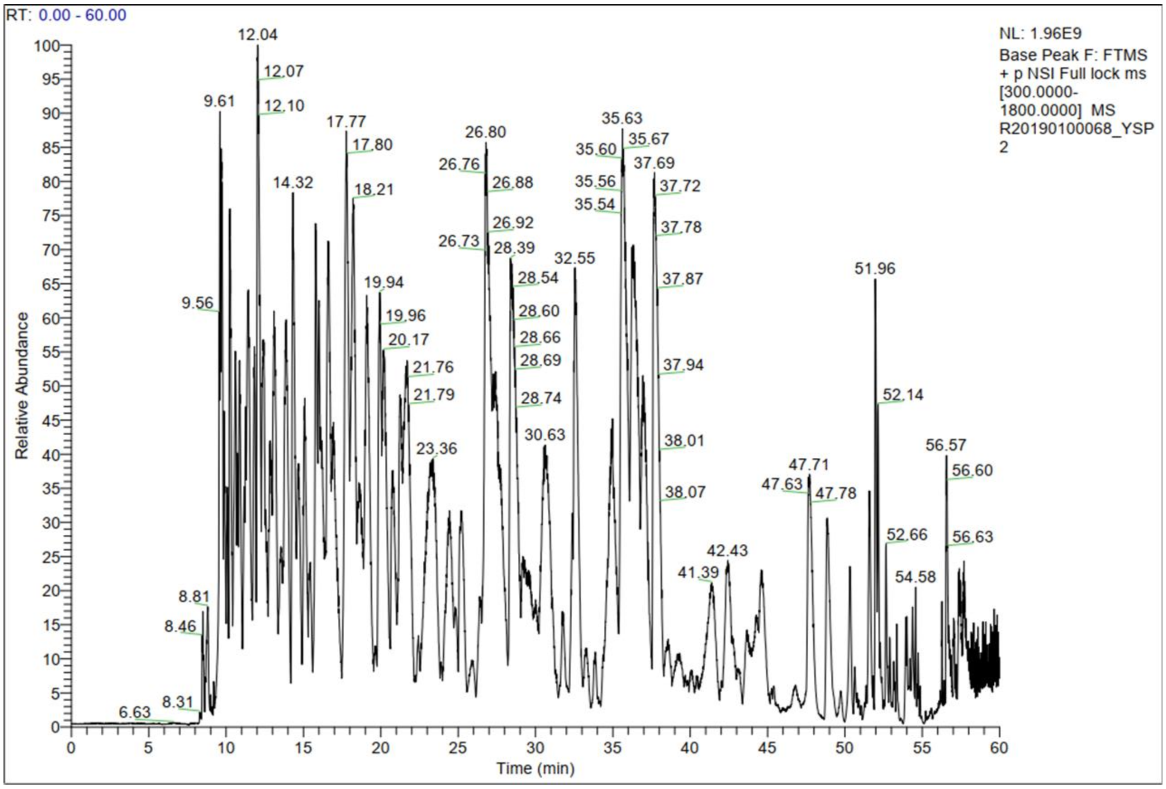

Figure S2: Base peak of phage YSP2 protein spectrum

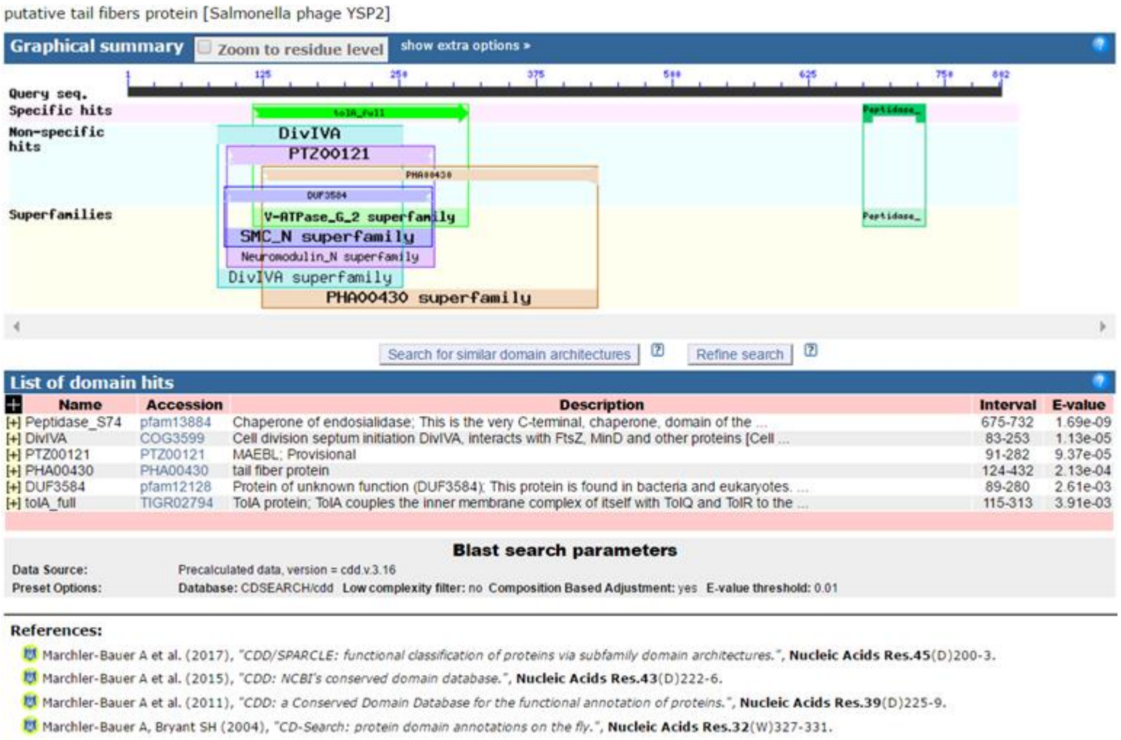

Figure S3: ORF35 amino acid function analysis

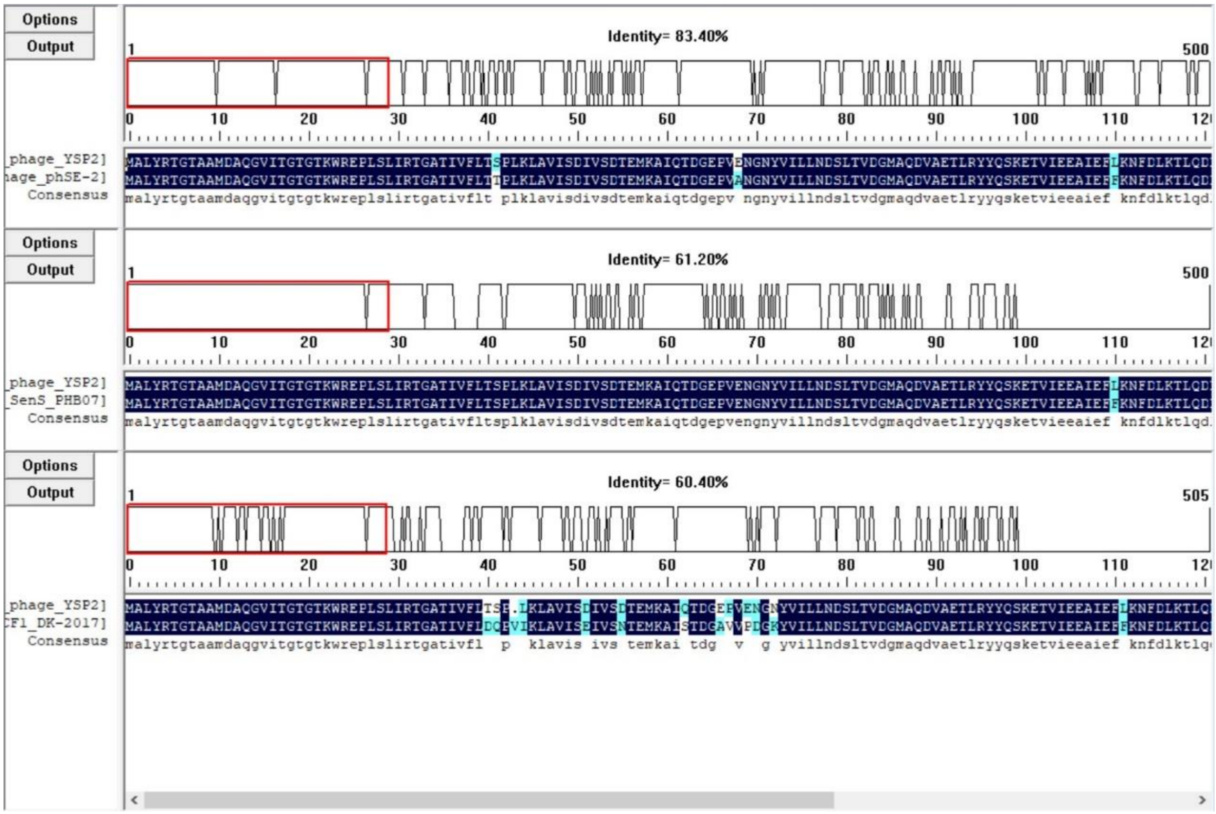

Figure S4: 35Q amino acid alignment analysis
